# The Antidepressant Mirtazapine Rapidly Shifts Hepatic B Cell Populations and Functional Cytokine Signatures in the Mouse

**DOI:** 10.3389/fimmu.2021.622537

**Published:** 2021-03-25

**Authors:** Wagdi Almishri, Rachelle P. Davis, Abdel-Aziz Shaheen, Mohammed O. Altonsy, Craig N. Jenne, Mark G. Swain

**Affiliations:** ^1^Department of Microbiology, Immunology, and Infectious Diseases, University of Calgary, Calgary, AB, Canada; ^2^Snyder Institute for Chronic Diseases, University of Calgary, Calgary, AB, Canada; ^3^Department of Pathology and Clinical Pathology, Faculty of Veterinary Medicine, University of Tripoli, Tripoli, Libya; ^4^Division of Gastroenterology and Hepatology, Department of Medicine, University of Calgary, Calgary, AB, Canada; ^5^Department of Zoology, Faculty of Science, Sohag University, Sohag, Egypt

**Keywords:** liver disease, immunity, B cells, inflammation, intravital microscopy

## Abstract

**Introduction:**

B cells are important regulators of both adaptive and innate immunity. The normal liver contains significant numbers of B cells, and their numbers increase dramatically in immune-mediated liver diseases. Our previous observations suggest a hepatoprotective effect of the antidepressant mirtazapine in human and experimental immune-mediated liver disease. Therefore, we performed a series of experiments to determine the impact of mirtazapine treatment on hepatic B cell homeostasis, as reflected by B cell number, trafficking and phenotype using flow cytometry (FCM) and intravital microscopy (IVM) analysis. Mirtazapine treatment rapidly induced a significant reduction in total hepatic B cell numbers, paralleled by a compositional shift in the predominant hepatic B cell subtype from B2 to B1. This shift in hepatic B cells induced by mirtazapine treatment was associated with a striking increase in total hepatic levels of the chemokine CXCL10, and increased production of CXCL10 by hepatic macrophages and dendritic cells. Furthermore, mirtazapine treatment led to an upregulation of CXCR3, the cognate chemokine receptor for CXCL10, on hepatic B cells that remained in the liver post-mirtazapine. A significant role for CXCR3 in the hepatic retention of B cells post-mirtazapine was confirmed using CXCR3 receptor blockade. In addition, B cells remaining in the liver post-mirtazapine produced lower amounts of the proinflammatory Th1-like cytokines IFNγ, TNFα, and IL-6, and increased amounts of the Th2-like cytokine IL-4, after stimulation *in vitro*.

**Conclusion:**

Mirtazapine treatment rapidly alters hepatic B cell populations, enhancing hepatic retention of CXCR3-expressing innate-like B cells that generate a more anti-inflammatory cytokine profile. Mirtazapine-induced hepatic B cell shifts could potentially represent a novel therapeutic approach to immune-mediated liver diseases characterized by B cell driven pathology.

## Highlights

Depression is common and often treated with antidepressants. However, how antidepressants alter immunity remains poorly understood. B cells are white blood cells that regulate how our immune system controls diseases, including liver disease. We found that treatment of mice with the widely used antidepressant mirtazapine causes a rapid shift in the B cell population within the liver, from a more pro-inflammatory type to a type that limits inflammation. This shift was in turn linked to decreased B cell patrolling within the blood vessels in the liver and through them sticking to other immune cells within the liver that also regulate liver immune responses. These findings suggest that mirtazapine can change how our liver responds to infections or other causes of injury and may contribute to improved outcomes in patients with liver disease.

## Introduction

Patients with various chronic health conditions, including those with chronic liver disease (CLD), have a higher prevalence of depression compared with age- and gender-matched healthy controls, greatly reducing their health-related quality of life (HRQOL) (1–3). This common comorbidity has prompted the investigation of various aspects of this association, most importantly, the role of immunity in the pathogenesis of depression (4, 5), and the potential effect of prescribed antidepressants on the immune system (6). A growing body of evidence indicates that many of the commonly prescribed antidepressants possess immunomodulatory properties, including altering plasma cytokine levels, changing immune cell numbers, impacting the proliferation abilities of immune cells, altering membrane transporters in immune cells, and changing regulatory immune cell populations (7–9). Given the strong association between depression and CLD, we assessed the impact of antidepressant treatment on CLD outcomes. We identified that the atypical antidepressant mirtazapine, alone among antidepressant subtypes, exhibited a significant protective effect in patients with the autoimmune liver disease primary biliary cholangitis (PBC), including a substantial decrease in mortality, decompensated cirrhosis, and liver transplantation (10).

Mirtazapine is an atypical antidepressant used primarily to treat major depressive disorder, but is also prescribed to treat a number of other conditions including anorexia, anxiety, nausea and vomiting, Parkinsonian tremor, and insomnia (9). Mirtazapine is presumed to exert its’ biological effect through antagonist effects on multiple receptor subtypes, including norepinephrine (α2 adrenergic), serotonin (5HT2c; 5HT2a, 5HT3) and histamine (H1) receptors (11). Importantly, serotonin receptors and histamine receptors are widely expressed on various immune cells, and immunoregulatory functions have been recognized for both serotonin and histamine, including modulation of cytokine secretion, immune cell maturation and activation, antigen presentation, humoral immunity, and recruitment (12, 13). Although evidence exists for an immunomodulatory effect of mirtazapine, few mechanistic studies have looked at the impact of mirtazapine on liver immunity. El-Tanbouly et al. showed that mirtazapine suppressed the progression of liver fibrosis in a mouse model (14), and we recently identified that mirtazapine altered hepatic innate immune responses and suppressed immune-mediated liver injury in a mouse model (15).

B cells are found in the healthy human liver where they account for up to 8% of the total hepatic lymphocyte population. Several hepatic B cell subpopulations are known to exist, including classical antibody producers, B2 B cells, and the innate-like B1 B cells (16, 17). Hepatic B cell numbers expand considerably in the context of most CLD’s, and conventionally have been implicated in disease pathogenesis (18). However, it is now appreciated that B cells can play opposing roles in regulating diseases, with different B lymphocyte subsets functioning as both regulators and inducers of immune responses (19–21). The recent identification of distinct B cell cytokine secreting populations that can be polarized to secrete Th1-like cytokines (B effector 1; Be1) or Th2-like cytokines (B effector 2; Be2) further highlights the importance of B cells as modulators of immune responses (22, 23). A number of reports have demonstrated that B lymphocytes can express functional receptors impacted by mirtazapine, including H1, 5HT3, and 5HT2A receptors (12, 24, 25). In addition, mirtazapine treatment could also potentially impact hepatic B cells indirectly, by interacting with receptors expressed on other immune cell populations within the liver, leading to shifts in cytokine release profiles and modulating the overall immune microenvironment. Therefore, these observations, combined with our previous findings, prompted us evaluate the potential effect of mirtazapine on the hepatic B cell population.

## Materials and Methods

### Animals and Experimental Treatments

Wild type 8–10-week-old male C57BL/6 mice (Jackson Labs, Bar Harbor, Maine), CD19-ZsGreen or CD19-tdTomato reporter mice were used. All animals were treated according to the criteria outlined in the ‘‘Guide for the Care and Use of Laboratory Animals’’ prepared by the National Academy of Sciences and published by the National Institutes of Health (NIH publication 86-23 revised 1985). All procedures were approved by the University of Calgary Animal Care Committee (#AC18-0154) and performed as per the guidelines of the Canadian Council on Animal Care. The fewest number of mice were used in each experiment to ensure scientific validity and were determined based on our significant previous work with this mouse model and techniques used (15, 26, 27). The number of animals used for individual experiments are reported in corresponding figure legends. To delineate the impact of mirtazapine on hepatic B cell homeostasis, we treated mice with a single injection of mirtazapine 20 mg/kg suspended in 1% aqueous solution of Tween80^®^ or vehicle intraperitoneally (IP; dose optimized in our previous studies) (15); animals were studied 3-5 hrs later.

#### Antibodies and Other Reagents

The following reagents and antibodies were obtained from indicated sources: Percoll^®^ (GE HealthCare Biosciences, Baue D’urfe, Quebec, Canada), dimethyl sulfoxide, TWEEN^®^ 80 and protease inhibitor cocktail (Sigma-Aldrich, St. Louis, MO), RPMI 1640 medium, HEPES, fetal bovine serum (FBS), and phosphate-buffered saline, Non-Essential Amino Acids Solution (100X), L-glutamine (200 mM), sodium pyruvate (100 mM), penicillin-streptomycin (10,000 U/mL) (Invitrogen, Life Technologies, Carlsbad, USA). Mirtazapine (CAS No: 85650-52-8; Tocris Bioscience, Bristol, UK). V-PLEX Proinflammatory Panel 1 (mouse) Kit (K0081381) (Meso Scale Diagnostics LLC, MD, USA). BD Cytofix/Cytoperm and the BD Perm/Wash, (BD, ON, Canada). Mouse CD19 Positive Selection Kit II (STEMCELL Technologies Canada Inc. BC, Canada). Anti-mouse CD11b (M1/70), anti-mouse Ly-6G (1A8), anti-mouse CD11c (N418), anti-mouse CD19 (6D5), anti-mouse CXCR3 (CXCR3-173), anti-mouse CD45 (30-F11), anti-mouse CD5 (53-7.3) (Biolegend, CA U.S.A). Anti-mouse CD16/CD32, anti-mouse IgM (eB121-15F9), anti-mouse CD3 (145-2C11), anti-mouse Ly6C (HK1.4), anti-mouse B220 (RA3-6B2), (eBioscience. San Diego, CA, USA). BCA Protein Assay kit (23227) (Pierce, USA). Anti-mouse CXCL10/IP-10 (6D4) (Novus Biologicals, Colorado, USA). To investigate the potential involvement of the chemokine receptor CXCR3 in hepatic B cell subset changes, an anti-CXCR3 neutralizing monoclonal antibody (clone CXCR3-173; 250μg/mouse), or Armenian hamster IgG isotype control (BioXcell), were administered intravenously (iv) 1 h before mirtazapine administration.

#### Liver Sample Preparation and Processing

*(i) Isolation of hepatic immune cells*: At specified time points post-treatment, mice were anesthetized (isoflurane; 2%, v/v in O2) and livers were perfused with 20 ml ice-cold PBS (19). The entire liver was removed and rinsed in cold-sterile PBS and underwent mechanical dissection, followed by density gradient centrifugation in (35% over 70%), as described previously (19) to isolate hepatic immune cells.

*(ii) Preparation of liver homogenate:* To assess hepatic chemokine (CXCL10) levels, the liver was perfused with 20 ml of ice-cold PBS, followed by 3ml of buffer containing protease inhibitors. The whole liver was then removed, cleaned, and homogenized in 2 ml of buffer containing protease inhibitors, centrifuged, passed through a 0.45-micron filter, and the homogenate stored at -20˚C (15).

### Flow Cytometry and Gating Strategies

Isolated hepatic leukocytes labelled using multicolor flow cytometry staining, as previously described (19). Cells were incubated with anti-CD16/CD32 to block non-specific binding to Fc III/II receptors followed by a wash step and subsequent incubation with conjugated antibodies to cell surface markers. For intracellular cytokine detection of CXCL10, cells were stained with antibodies to cell surface antigens, fixed and permeabilized with the BD Cytofix/Cytoperm, and stained with conjugated anti-CXCL10. Samples were acquired either using Attune™ Acoustic Focusing flow cytometer (Applied Biosystems, Ontario, CA) or Cytoflex LX (Beckman Coulter, California, USA). Data were analyzed using FlowJo^®^ software (Treestar, OR, USA). Gating proceeded as follows: After doublet exclusion, gating on forward scatter (FSC-A) and side scatter (SSC-A) parameters was set to include all leukocytes and exclude cell debris. B cells were identified as CD3^-^IgM^+^, B-2 B cells as (CD5^-^CD11b^-^) and B1a B cells as CD11b^+^CD5^+^ (28–30). Monocytes were identified as CD11b^+^Ly6G^-^Ly6C^+^ (15) and dendritic cells as CD11b^+^CD11c^+^ (31). Fluorescence-minus-one (FMO) controls were used for the accurate designation of cells with fluorescence above background levels (15). Cell numbers were calculated based on the percentage of cells found in the gate of interest and the total numbers isolated from each liver.

### Assessment of Cytokine and Chemokine Levels

Levels of the CXCR3-chemokine ligand CXCL10 were measured in liver homogenates by Luminex^®^ (Eve Technologies Corporation, Calgary, Canada). Liver homogenate protein concentrations were quantified using a BCA Protein Assay kit (Pierce, USA) and results expressed as pg/mg protein (15). To measure CXCL10 production from myeloid cells, enriched mouse peritoneal macrophages were cultured with mirtazapine or vehicle *in vitro* for 24 hrs. Murine peritoneal macrophages were obtained following injecting of naïve C57Bl/6 mice with a 4% thioglycolate, as described previously (32), and seeded into 24-well tissue culture plates (density of 1 × 10^6^ cells/well) in 500 μl RPMI 1640 medium supplemented with 10% FBS, 1 mM sodium pyruvate, 2 mM L-glutamine, and 100 units/ml penicillin and streptomycin, and non-essential amino acids (NEAA). Cell were treated with mirtazapine (10 μM) (15), or vehicle (0.2% DMSO), and cultured for 24 h. Supernatants were collected, and CXCL10 levels in cell culture supernatants were measured by Luminex^®^ (Eve Technologies Corporation, Calgary, Canada).

To determine B cell cytokine secretion profiles, freshly isolated single-cell suspensions of hepatic leukocytes were prepared from the livers of mice at 5 hrs post-mirtazapine or vehicle treatment. and were enriched for B cells using a CD19 Positive Selection Kit II (STEMCELL Technologies Canada Inc. BC, Canada) (hepatic B cell purity was confirmed to be > 96%). B cells (2× 105 in 200 μl/well) were stimulated with a cocktail of phorbol 12-myristate 13-acetate (PMA) and ionomycin (50ng/ml and 1 μg/ml, respectively; eBioscience) for 24 h in complete RPMI-1640 media (supplemented with 10% fetal calf serum, nonessential amino acids, L-glutamine, β-mercaptoethanol, and penicillin-streptomycin; all reagents Invitrogen, Canada). Cell culture supernatants were harvested and analyzed for cytokines using V-PLEX Proinflammatory Panel 1 Mouse Kit (K0081381, Meso Scale Diagnostics, Rockville, Maryland) according to manufacturer’s instructions. The multiplexing analysis was performed using the MESO QuickPlex SQ 120 system (Meso Scale Diagnostics).

### Liver Intravital Microscopy and Analysis

Surgical preparation of animals for liver intravital microscopy was performed as previously described (33). B cells were visualized with the use of CD19-ZsGreen or CD19-tdTomato reporter mice, or by labelling with Brilliant Violet 421- conjugated anti-mouse CD19 (6D5) antibodies (Biolegend). For CXCR3 blockade experiments, anti-CXCR3 (clone CXCR3-173; 250μg/mouse) was administered 1hr before mirtazapine (or vehicle), and images were acquired 4-5 hrs post-mirtazapine. Kupffer cells were visualized using an Alexa Fluor 488-conjugated anti-mouse F4/80 (BM8) antibody (Biolegend). Quantification of B cells/field of vision (FOV), and CD19^+^/F4/80^+^ contact interactions, were done using LasX analysis software; an interaction was defined as a CD19^+^ cell positioned < 2µm away from F4/80+ cell for >30 seconds. For sinusoidal diameter calculations, ten sinusoids/FOV were measured *via* LasX software and averaged to determine the mean sinusoid diameter for that FOV (3 FOV/animal, 5-6 animals/group). Sinusoidal particle speed was calculated as fluorescent bead distance traveling through 80-120 µm of sinusoid/seconds, from start to end of a given distance (5 measurements/animal, 5-6 animals/group). The flow rate was calculated by multiplying velocity by sinusoidal area, where area= πr^2^ (r = radius of sinusoid; 5 measurements/animal, 5-6 animals/group). B cell behavior was analyzed using LasX software; every CD19^+^ cell in 3 FOV/animal was analyzed in videos >5 min to assess distance traveled and cell velocity. For B cell migration plots, X/Y position was normalized to a reference point (0,0), and each cell’s X/Y trajectory position was plotted for each time point. Every cell/FOV was tracked for 10 mins (2 randomly selected FOVs per treatment group). Resulting trajectories were plotted as a representative schematic using GraphPad Prism (version 8.4.3).

### Statistical Analyses

All data are shown as mean ± standard error of the mean (SEM). For comparisons between two groups, an unpaired Student’s t-test was used. For comparisons between more than two groups an analysis of variance followed by the Student-Newman-Keuls post-hoc test was performed (Graph-Pad V8.4.3, San Diego, CA).

## Results

### Mirtazapine Treatment Induces a Rapid Reduction in Intrahepatic B Cell Numbers and Shifts the Hepatic B1a and B2 Subset Profile

**Figure 1:**
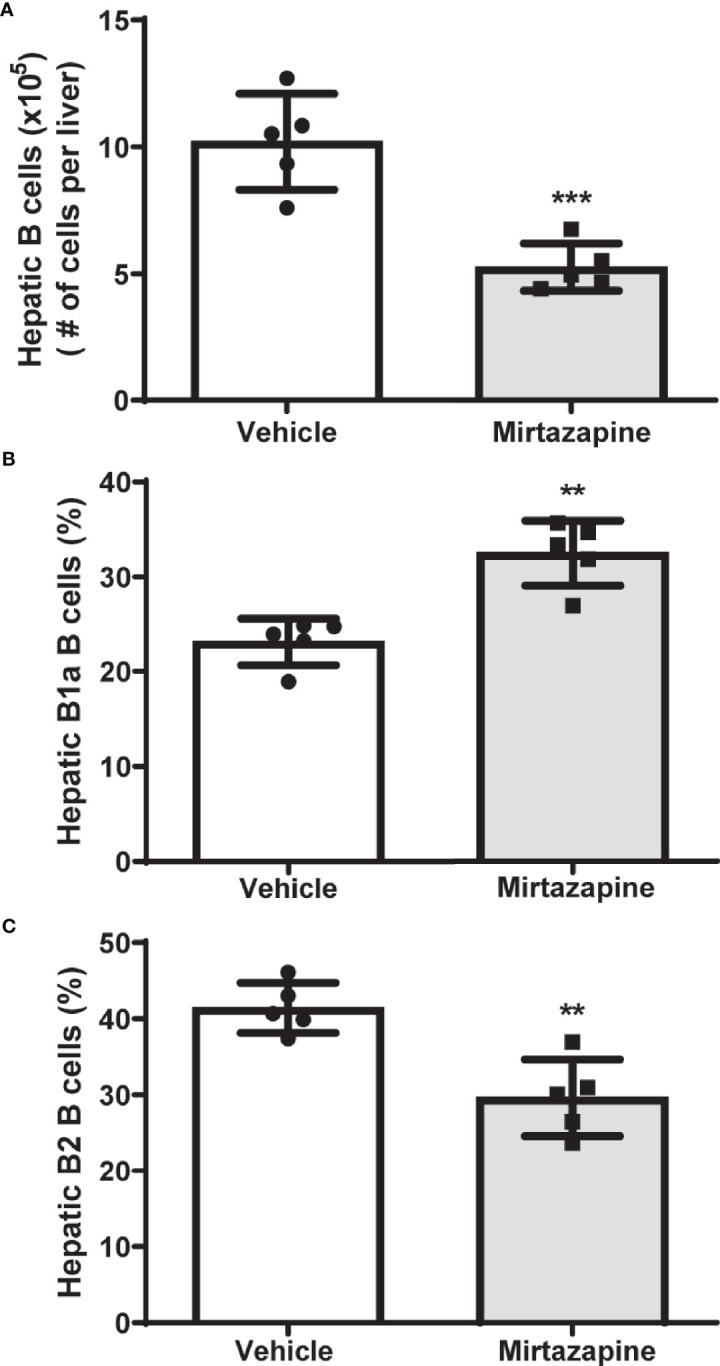
Mirtazapine treatment significantly decreases hepatic B cell numbers and induces compositional changes in hepatic B cell subtypes. **(A)** Administration of mirtazapine resulted in ~ 2-fold reduction in numbers of hepatic B cells 5 hrs post-Mirtazapine administration vs vehicle treated mice (***p ≤0.0008 n = 5 mice/group). **(B)** B1a B cells are significantly enriched within the livers post-mirtazapine treatment (**p ≤0.001; n = 5 mice/group). **(C)** Hepatic B2 cells are significantly reduced in mirtazapine-treated vs. vehicle-treated treated mice (**p ≤0.004; n = 5 mice/group).

Using flow cytometric analysis, we found that mirtazapine treatment induced a significant decrease in hepatic B cell numbers, compared with vehicle-treated controls (**Figure 1A**). Moreover, this mirtazapine-induced reduction in hepatic B cells was not uniform among the major B cell subsets. Specifically, mirtazapine treatment led to a disproportionate enrichment of B1a B cells, and a parallel reduction in the B2 cell population within the liver (**Figures 1B, C**).

**Figure 2:**
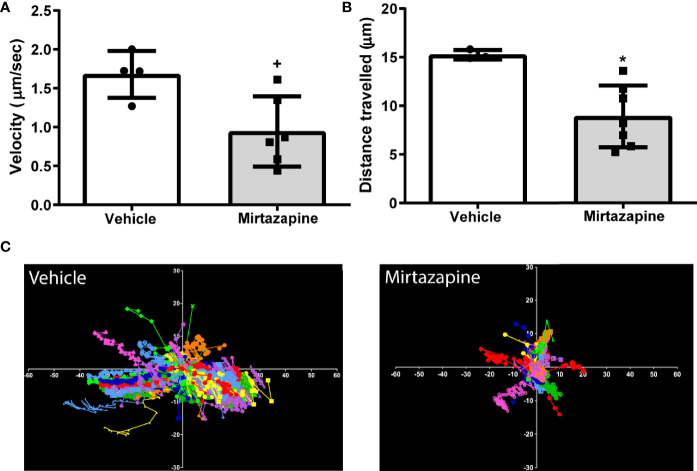
Reduced sinusoidal patrolling activity of hepatic B cells after mirtazapine treatment. Mice were treated with mirtazapine or vehicle, and cell mobility parameters such as velocity and total distance traveled (the cumulative length of the trajectory) per cell were quantified from analyses of live cell imaging results. **(A)** Mean movement velocity of hepatic B cells and **(B)** average distance traveled by hepatic B cells. Data are shown as mean +/- SEM, Student’s t test ^+^p ≤ 0.05, *p ≤ 0.02 n = 4/7mice/group. **(C)** Representative plots of B cell motion trajectories from one field of view of vehicle or mirtazapine treated livers. Each vector represents displacement of an individual B cell for an imaging period of >5 min relative to their starting position.

We speculated that the rapid decrease in overall hepatic B cell numbers may be due to a mirtazapine-related increase in sinusoidal blood flow driving non-adherent, or poorly adherent, B cells from the liver. Indeed, using IVM we found that mirtazapine treatment-induced an increase in the hepatic sinusoidal blood volumetric flow rate, associated with a significant increase in hepatic sinusoid diameter, compared to vehicle-treated control mice. In contrast, the velocity of intravenously administered particles within the hepatic sinusoids was not altered by mirtazapine treatment (**Supplementary Figures 1A–D**). Following mirtazapine treatment, intravascular B cell migration (as measured by IVM) was significantly reduced, as reflected by reduced cellular velocity and reduced distance travelled by hepatic B cells in the mirtazapine treated group (**Figures 2A–C**).

To address possible mirtazapine-related B cell toxicity as a potential cause of reduced hepatic B cell numbers, whole mouse peripheral blood was incubated with mirtazapine or vehicle *in vitro* for 4 hrs. Cells were stained with annexin V and a cell viability dye and subsequently analyzed by flow cytometry. Mirtazapine did not induce direct apoptotic or cytotoxic effects on B cells (**Supplementary Figures 2A–C**).

### Mirtazapine Treatment Rapidly Increases Hepatic CXCL10 Levels and Upregulates CXCR3 Expression on Intrahepatic B Cells

**Figure 3:**
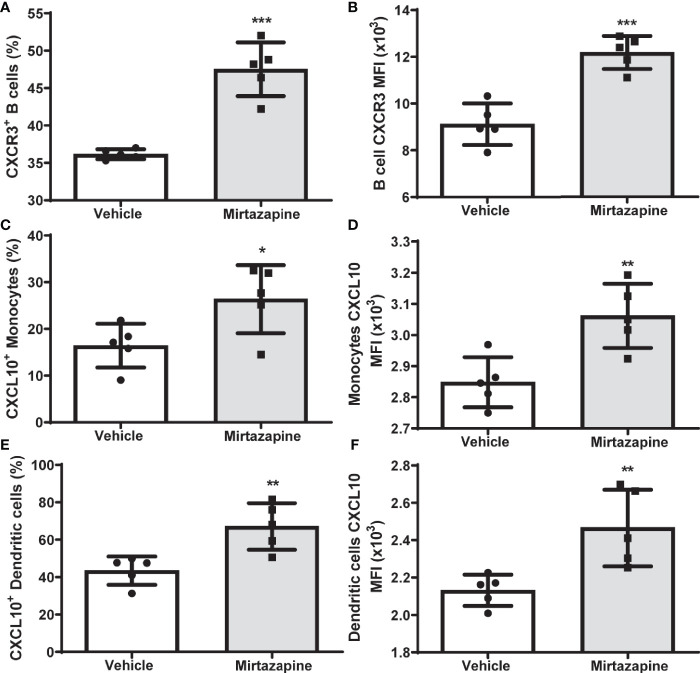
Upregulation of hepatic CXCL10 levels and increased proportions of CXCR3-expressing hepatic B cells after mirtazapine treatment. **(A)** Mirtazapine treatment significantly enriches hepatic CXCR3 expressing B cells 5hrs post- treatment, compared to vehicle-treated controls (***p ≤0.0001; n = 5 mice/group). **(B)** Hepatic B cells in mirtazapine-treated mice demonstrate a higher mean fluorescence intensity (MFI) for CXCR3 expression than B cells from vehicle-treated mice (***p ≤0.0003; n = 5 mice/group). Mirtazapine treatment increases production (MFI) and frequency (%) of CXCL10^+^ hepatic monocytes **(C, D)** and dendritic cells **(E, F)** *p ≤0.034 for **(C)**, (*p ≤0.0064 for **(D)**, **p ≤0.007 for **(E)**; and **p ≤0.0099 for **(F)**; n = 5 mice/group.

Flow cytometric analyses 5 hrs post-mirtazapine revealed a marked upregulation of CXCR3 expression on intrahepatic B cells reflected by an increase in both the percentage of CXCR3-positive B cells (**Figure 3A**) and an increase in CXCR3 expression per cell (**Figure 3B**). Macrophage derived CXCL10 has previously been implicated in the regulation of B cell phenotypic changes (34). Therefore, we determined the impact of mirtazapine treatment on hepatic CXCL10 levels. We found that mirtazapine treatment induced a significant increase in hepatic CXCL10 protein levels 5 hrs post-treatment (vehicle: 1.610 ± 0.081 pg/mg vs. mirtazapine: 192.5± 60.8 pg/mg; **p ≤ 0.004; n = 5 and 7 mice/group). Moreover, mirtazapine treatment significantly increased the proportion of CXCL10 expressing monocytes (**Figure 3C**) and dendritic cells (**Figure 3E**)), and increased CXCL10 expression (as mean fluorescence intensity; MFI) in hepatic monocytes (**Figure 3D**) and dendritic cells (**Figure 3F**), in the liver of mirtazapine treated mice compared to vehicle treated controls. Importantly, treatment of mouse macrophages with mirtazapine *in vitro* did not induce CXCL10 production, indicating mirtazapine does not directly stimulate macrophages to produce CXCL10 (vehicle: 6.9 ± 0.76 pg/ml vs. mirtazapine: 7.5 ± 0.89 pg/ml; ns [p ≤ 0.62]; n=4/group).

### CXCR3 Mediates the Preferential Retention of B Cells Within the Liver Following Mirtazapine Treatment

**Figure 4:**
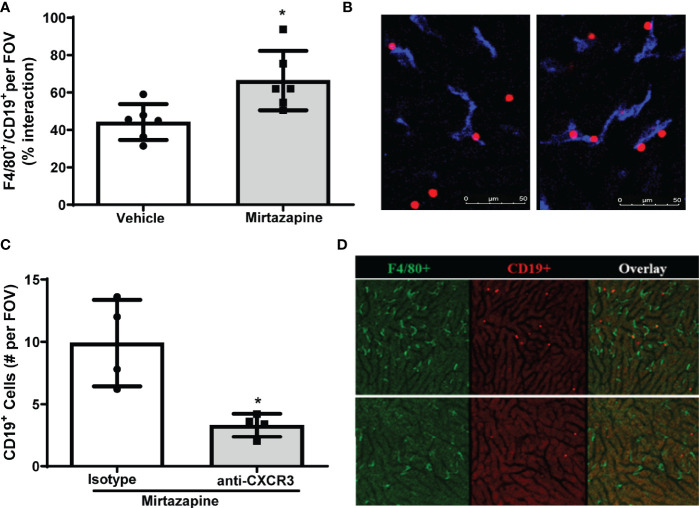
CXCR3 chemokine receptor contributes to preferential retention of B cells within the liver. **(A)** Quantification of F4/80^+^ and CD19^+^ cell interactions in livers 3 hrs. post-vehicle or mirtazapine treatment (*p ≤0.02; n = 6 mice/group). **(B)** Representative liver IVM image from a mouse 3 hrs post-vehicle (left) and 3 hrs post-mirtazapine treatment (right) showing enhanced intimate B cell (CD19^+^; red) and Kupffer cell (F4/80^+^; blue) interactions in mirtazapine vs vehicle treated mice. **(C)** Reduction in hepatic B cells in mirtazapine-treated mice after CXCR3 blockage. B cells (CD19^+^) were counted in at least 5 FOV images/mouse (*p ≤0.05 n = 4 mice/group). **(D)** Representative liver IVM images from mice 3 hrs. post-treatment. Mirtazapine + isotype control upper panels, and mirtazapine + anti-CXCR3 neutralizing antibody (lower panels). Green staining indicates F4/80^+^ Kupffer cells and red staining indicates CD19^+^ B cells.

To identify cellular interactions responsible for the retention of B cells within the liver post-mirtazapine treatment, we performed IVM analysis of hepatic B cell and Kupffer cell interactions post-mirtazapine treatment using CD19-tdTomato reporter mice (B cells appear “red” in IVM). Using IVM, we observed a significantly higher proportion of hepatic B cells in contact with Kupffer cells after mirtazapine treatment, compared to vehicle-treated mice (**Figures 4A, B**). Furthermore, *in vivo* antibody-mediated blockade of CXCR3 significantly reduced hepatic B cells within the liver post-mirtazapine treatment, compared to mirtazapine treated mice that received isotype control (**Figures 4C, D**). These observations indicate that CXCR3 plays an important role in differential B cell retention within the liver after mirtazapine treatment.

### The Hepatic B Cell Population Post-Mirtazapine Treatment Exhibits a Shift Towards Th2-Like Effector Cytokine Production

**Figure 5:**
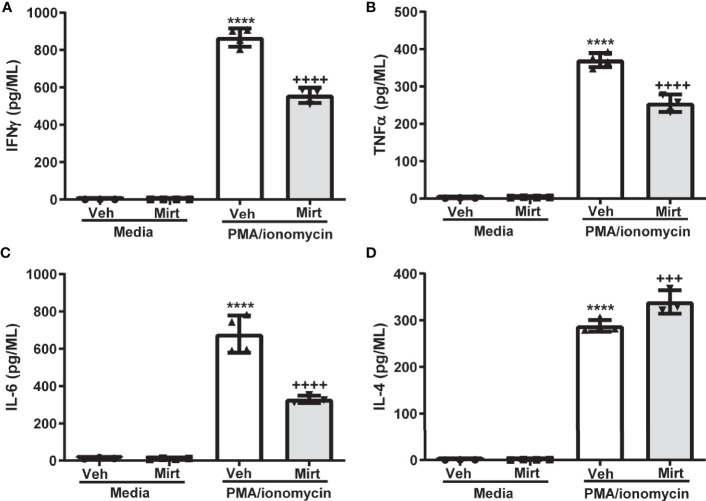
Mirtazapine alters the cytokine secreting profile of the hepatic B cell population. Purified hepatic B cells were isolated from vehicle or mirtazapine treated mice and stimulated *in vitro*) with PMA/ionomycin and cytokine release measured. Production of proinflammatory cytokines IFN-γ **(A)**, TNFα **(B)**, and IL-6 **(C)** were lower from the hepatic B cell population isolated from mirtazapine vs vehicle treated mice, whereas production of the anti-inflammatory cytokine IL-4 **(D)** was increased in the B cell population isolated from mirtazapine vs vehicle treated mice. **(A)** ****p ≤0.0001 vehicle + PMA/ionomycin *vs.* all other groups; ^++++^p ≤0.0001 mirtazapine +PMA/ionomycin *vs.* all other groups, **(B)** ****p ≤0.0001 vehicle + PMA/ionomycin *vs.* all other groups; ^++++^p ≤0.0001 mirtazapine + PMA/ionomycin *vs.* all other groups, **(C)** ****p ≤0.0001 vehicle + PMA/ionomycin *vs.* all other groups; ^++++^p ≤ 0.0001 mirtazapine + PMA/ionomycin *vs.* all other groups, and **(D)** ****p ≤0.0001 vehicle + PMA/ionomycin and mirtazapine + PMA/ionomycin groups *vs.* all other groups; and ^+++^p ≤0.001 vehicle + PMA/ionomycin *vs.* mirtazapine + PMA/ionomycin group (n = 3 and 4 mice per group).

The reduction in hepatic B cell numbers and skewing of the hepatic B1a: B2 B cell ratio induced by mirtazapine, prompted us to investigate whether B cells remaining within the liver post-mirtazapine also generated an altered cytokine profile. Indeed, we found that B cells remaining within the liver post-mirtazapine treatment secrete less proinflammatory cytokines after *in vitro* stimulation (i.e. IFNγ, TNF-α, and IL-6) coupled with enhanced IL-4 production (**Figures 5A–D**). In contrast, hepatic B cells isolated from mirtazapine treated mice and stimulated *in vitro* produced similar amounts of the cytokines IL-12p70, IL-5 and IL-10 as did B cells isolated from vehicle treated mice (**Supplementary Figures 3A–C**).

## Discussion

We examined the impact of the atypical antidepressant mirtazapine on hepatic B cells and found that administration of mirtazapine in mice profoundly impacts hepatic B cell homeostasis and effector function. In addition to its metabolic, secretory and excretory functions, the liver is also appreciated for its complex immunological activities facilitated by a diverse population of tissue-resident immune cells. In particular, B cells reside in the healthy liver, and an increase in hepatic B cell numbers has traditionally been implicated in the development of CLD (18). However, accumulating clinical and experimental evidence suggests that B cell-depleting reagents can also aggravate a number of diseases, including those affecting the liver (19, 21, 35, 36). These observations attest to functional heterogeneity of B cells. In this study, we observed a significant and rapid decrease in hepatic B cell numbers after mirtazapine treatment. Moreover, hepatic B cells that remained in the liver post-mirtazapine treatment were enriched in B1a B cells, with fewer B2 B cells, compared to hepatic B cell populations found in control mice. There is a growing body of literature ascribing a protective role for the innate-like B1a B cell subset in many diseases (21, 37, 38). However, studies exploring the role of the B1a B cell subset in the context of liver disease are scarce. An elegant study by Yang et al. implicated dysfunctional B1a cells and associated defective regulatory functions in the pathogenesis of a murine model of primary biliary cholangitis (PBC) (39). Thus, tipping the balance of the B-cell pool towards more innate-like effector-regulatory B cells could be a potential mechanism through which mirtazapine exerts a protective effect in the context of a wide array of human immune-mediated illnesses, including the liver disease PBC, as we have documented in epidemiological studies from our group (10, 40).

Chemokine-receptor/ligand interactions have been identified as critical recruitment and retention signals for immune cells, including B cells (41–44). We found a striking upregulation of CXCR3 on the surface of hepatic B cells, and an increased proportion of CXCR3-expressing B cells in the B cell pool retained within the liver post-mirtazapine treatment. In keeping with previous observations (34), we speculated that the mirtazapine-related up-regulation of CXCR3 in hepatic B cells might promote preferential retention of different B cell subsets within the liver through interactions with its’ ligand CXCL10, following mirtazapine treatment. Therefore, we determined changes in hepatic expression of CXCL10 post-mirtazapine treatment. Indeed, hepatic CXCL10 levels increased significantly after mirtazapine therapy. Our flow cytometry data showed that in normal liver immune cells CXCL10 is expressed primarily by hepatic macrophages and dendritic cells. Moreover, a significant increase in CXCL10 expression by these immune cells was noted at 5 hours post-mirtazapine treatment, a finding further implicating a potential role for CXCR3: CXCL10 interactions in the differential hepatic B cell retention we had observed post-mirtazapine. The effect of mirtazapine on CXCL10 production by macrophages appears to be indirect since the treatment of macrophages with mirtazapine *in vitro* did not induce CXCL10 production. We next investigated the functional role of CXCR3 in hepatic B cell retention by administrating an anti-CXCR3 neutralizing antibody prior to mirtazapine treatment. CXCR3 blockade significantly reduced hepatic B cell numbers in mirtazapine treated mice compared to isotype treated controls receiving mirtazapine, providing clear evidence for a functional role of CXCR3 in mediating B cell retention within the liver post-mirtazapine treatment. Consistent with our findings, CXCR3 has been previously implicated in non-B lymphocyte immune cell retention in the liver (45). Furthermore, it has been established that CXCR3 ligands can trigger integrin-mediated adhesion of CXCR3-expressing hepatic and peripheral blood lymphocytes to integrins (46, 47).

Increased liver sinusoidal diameter and increased sinusoidal blood volumetric flow rate were both observed in the mirtazapine-treated group, compared to the vehicle control group, suggesting a role for mirtazapine-related receptor blockade in the regulation of sinusoidal hemodynamics. Indeed, it has been previously shown that serotonin reduces hepatic sinusoidal blood flow, an effect that was inhibited by 5-HT2 receptor antagonists (48). Therefore, it is plausible that by antagonizing endogenous serotonin induced 5-HT2 -mediated receptor activity in the liver, mirtazapine causes sinusoidal dilation, as indicated by increased sinusoidal diameter. In addition to overall blood flow, these alterations to hemodynamic parameters are anticipated to impact the retention and behavior of intravascular leukocytes within the liver. We speculate that the increased hepatic sinusoidal volumetric flow rate that occurred post-mirtazapine treatment caused the removal of non- or poorly-adherent B cells from the liver, leaving behind B cells that were firmly anchored by enhanced interactions with either the sinusoidal endothelium or Kupffer cells within the liver, following mirtazapine treatment. Indeed, mirtazapine treatment rapidly induced changes in hepatic B cell motility patterns, as reflected by reduced speed of movement and decreased distance travelled. These alterations in B cell movement suggest reduced patrolling of the liver vasculature in mirtazapine-treated animals. Moreover, mirtazapine induced an increase in hepatic B cell – Kupffer cell adhesive interactions *in vivo*, mediated by B cell expression of the chemokine receptor CXCR3, further enhancing the retention of a specific population of hepatic B cells.

The pronounced effect of mirtazapine on hepatic B cell hemostasis was associated with a significant alteration in hepatic B cell cytokine effector function. Hepatic B cells isolated from vehicle or mirtazapine treated mice produced comparable baseline levels of cytokines in the absence of stimulation. However, the B cell pool retained within the liver post-mirtazapine treatment produced decreased amounts of several proinflammatory cytokines, including IFNγ, TNFα and IL-6, and enhanced secretion of the anti-inflammatory cytokine IL-4, after *in vitro* stimulation, compared to the hepatic B cell pool isolated from vehicle-treated mice. Cytokines are crucial pathophysiological regulators of acute and chronic liver diseases, and there has been considerable interest in developing therapeutic agents that can modulate expression of specific cytokines necessary for the regulation of hepatic inflammation and repair (49). In the context of liver diseases, cytokines have been assigned fundamental properties reflecting common function, including proinflammatory cytokines (e.g. IL‐1α, IL‐1β, TNF‐α, and IL‐2), immunoregulatory cytokines (e.g. IFN-γ), and anti-inflammatory cytokines (e.g. IL-10 and IL-4) (49). In contrast, IL-6 has a less well defined role in the context of inflammatory liver disease, in that it has been ascribed both pro‐ and anti‐inflammatory activities (50). Based on their conventionally reported roles, our data suggest that mirtazapine favors skewing of the B cell-related intrahepatic cytokine milieu towards a more anti-inflammatory/immune-suppressive one. If a similar change occurs in patients receiving mirtazapine, this hepatic cytokine shift may alter immune responses away from a more proinflammatory profile.

Although serotonin appears to be capable of modulating B cell responses, this linkage remains somewhat controversial in some models (51). Specifically, serotonin upregulates mitogen-stimulated B lymphocyte proliferation, an effect mediated through the 5-HT1A receptor subtype (52). Mirtazapine is considered an antagonist of 5HT2A, 5HT2C and 5HT3 serotonin receptors, not 5HT1A receptors (53). Moreover, B cells express 5HT2A and 5HT3 receptors (54, 55).We have previously reported that mirtazapine treatment does not alter total hepatic serotonin levels (15, 27). However, mirtazapine treatment may potentially alter hepatic B cell responses by blocking signaling through 5HT2A, 5HT2C and 5HT3 serotonin receptor subtypes, or by augmenting signaling through other serotonin receptor subtypes (including 5HT1A) by enhancing the local availability of serotonin (11). Additionally, mirtazapine could potentially impact other innate or adaptive immune cell types within the liver that express serotonin receptors and modulate their activation and cytokine secretion to subsequently alter hepatic B cell immune responses.

Here, we report the identification of a novel immunomodulatory effect of mirtazapine on hepatic B cells, skewing the hepatic B cell population towards a more B1 dominant profile commonly associated with a more regulatory and/or anti-inflammatory phenotype (21, 37, 38, 56). Our current findings are consistent with our previously published results showing that mirtazapine exerts anti-inflammatory effects within the liver in a mouse model of immune-mediated hepatitis, and our epidemiological findings showing a beneficial effect of mirtazapine on adverse hepatic outcomes in patients with the liver disease PBC (10, 15). Given the growing interest in identifying innovative therapies that can be used as an alternative to pan-B cell-depleting treatments, mirtazapine, or mirtazapine derivative, could potentially represent a novel therapeutic strategy directed towards enriching hepatic immune-regulatory B1a lymphocytes, and reducing pathogenic B2 lymphocytes to beneficially alter immune-mediated liver disease outcomes.

## Data Availability Statement

The original contributions presented in the study are included in the article/**Supplementary Material**. Further inquiries can be directed to the corresponding author.

## Ethics Statement

The animal study was reviewed and approved by Health Sciences Animal Care Committee; University of Calgary.

## Author Contributions

WA and RD: conceived, designed, performed experiments, analyzed the data, prepared figures, and wrote the manuscript. CJ and MS: conceived, designed, and supervised the research, analyzed data, and wrote the manuscript. A-AS: conceived and contributed to the generation of the manuscript. MA: contributed to the generation of the manuscript. All authors contributed to the article and approved the submitted version.

## Funding

This work was supported by a Team Grant (Chronic Inflammation Initiative) awarded by the Canadian Institutes of Health Research (MS as principal investigator), and by the Cal Wenzel Family Foundation Chair in Hepatology (held by MS).

## Conflict of Interest

The authors declare that the research was conducted in the absence of any commercial or financial relationships that could be construed as a potential conflict of interest.

## Abbreviations

FCM, Flow cytometry; CLD, Chronic liver disease; HRQOL, Health-related quality of life; PBC Primary biliary cholangitis; MFI, Mean fluorescence intensity; IVM, intravital microscopy; FMO, Fluorescence-minus-one; FOV, Field of view; PMA, phorbol myristate acetate.

## References

[1] Katon WJ. Epidemiology and treatment of depression in patients with chronic medical illness. Dialogues Clin Neurosci (2011) 13(1):7–23. 10.31887/DCNS.2011.13.1/wkaton 21485743 PMC3181964

[2] Huang X Liu X Yu Y. Depression and Chronic Liver Diseases: Are There Shared Underlying Mechanisms? Front Mol Neurosci (2017) 10:134. 10.3389/fnmol.2017.00134 28533742 PMC5420567

[3] Graff LA Walker JR Bernstein CN. Depression and anxiety in inflammatory bowel disease: a review of comorbidity and management. Inflammation Bowel Dis (2009) 15(7):1105–18. 10.1002/ibd.20873 19161177

[4] D’Mello C Swain MG. Immune-to-Brain Communication Pathways in Inflammation-Associated Sickness and Depression. Curr Top Behav Neurosci (2017) 31:73–94. 10.1007/7854_2016_37 27677781

[5] Leonard BE. The concept of depression as a dysfunction of the immune system. Curr Immunol Rev (2010) 6(3):205–12. 10.2174/157339510791823835 PMC3002174 21170282

[6] Szałach ŁP Lisowska KA Cubała WJ. The Influence of Antidepressants on the Immune System. Archivum immunologiae therapiae experimentalis (2019) 67(3):143–51. 10.1007/s00005-019-00543-8 PMC6509093 31032529

[7] Strawbridge R Arnone D Danese A Papadopoulos A Herane Vives A Cleare AJ. Inflammation and clinical response to treatment in depression: A meta-analysis. Eur Neuropsychopharmacol (2015) 25(10):1532–43. 10.1016/j.euroneuro.2015.06.007 26169573

[8] Dahl J Ormstad H Aass HC Malt UF Bendz LT Sandvik L. The plasma levels of various cytokines are increased during ongoing depression and are reduced to normal levels after recovery. Psychoneuroendocrinology (2014) 45:77–86. 10.1016/j.psyneuen.2014.03.019 24845179

[9] Peña S Baccichet E Urbina M Carreira I Lima L. Effect of mirtazapine treatment on serotonin transporter in blood peripheral lymphocytes of major depression patients. Int Immunopharmacol (2005) 5(6):1069–76. 10.1016/j.intimp.2005.02.005 15829422

[10] Shaheen AA Kaplan GG Almishri W Vallerand I Frolkis AD Patten S. The impact of depression and antidepressant usage on primary biliary cholangitis clinical outcomes. PloS One (2018) 13(4):e0194839. 10.1371/journal.pone.0194839 29617396 PMC5884515

[11] Anttila SAK Leinonen EVJ. A Review of the Pharmacological and Clinical Profile of Mirtazapine. CNS Drug Rev (2001) 7(3):249–64. 10.1111/j.1527-3458.2001.tb00198.x PMC6494141 11607047

[12] Herr N Bode C Duerschmied D. The Effects of Serotonin in Immune Cells. Front Cardiovasc Med (2017) 48(48):1–11. 10.3389/fcvm.2017.00048 PMC5517399 28775986

[13] Branco ACCC Yoshikawa FSY Pietrobon AJ Sato MN. Role of Histamine in Modulating the Immune Response and Inflammation. Mediators Inflammation (2018) 2018:9524075–. 10.1155/2018/9524075 PMC6129797 30224900

[14] El-Tanbouly DM Wadie W Sayed RH. Modulation of TGF-β/Smad and ERK signaling pathways mediates the anti-fibrotic effect of mirtazapine in mice. Toxicol Appl Pharmacol (2017) 329:224–30. 10.1016/j.taap.2017.06.012 28623179

[15] Almishri W Shaheen AA Sharkey KA Swain MG. The Antidepressant Mirtazapine Inhibits Hepatic Innate Immune Networks to Attenuate Immune-Mediated Liver Injury in Mice. Front Immunol (2019) 10:803(803). 10.3389/fimmu.2019.00803 31031775 PMC6474187

[16] Robinson MW Harmon C O’Farrelly C. Liver immunology and its role in inflammation and homeostasis. Cell Mol Immunol (2016) 13(3):267–76. 10.1038/cmi.2016.3 PMC4856809 27063467

[17] Norris S Collins C Doherty DG Smith F McEntee G Traynor O. Resident human hepatic lymphocytes are phenotypically different from circulating lymphocytes. J Hepatol (1998) 28(1):84–90. 10.1016/s0168-8278(98)80206-7 9537869

[18] Taylor SA Assis DN Mack CL. The Contribution of B Cells in Autoimmune Liver Diseases. Semin liver Dis (2019) 39(4):422–31. 10.1055/s-0039-1688751 PMC6800599 31226726

[19] Almishri W Deans J Swain MG. Rapid activation and hepatic recruitment of innate-like regulatory B cells after invariant NKT cell stimulation in mice. J Hepatol (2015) 63(4):943–51. 10.1016/j.jhep.2015.06.007 26095178

[20] Liu Y Cheng LS Wu SD Wang SQ Li L She WM. IL-10-producing regulatory B-cells suppressed effector T-cells but enhanced regulatory T-cells in chronic HBV infection. Clin Sci (Lond) (2016) 130(11):907–19. 10.1042/cs20160069 26980345

[21] Thaunat O Morelon E Defrance T. Am”B”valent: anti-CD20 antibodies unravel the dual role of B cells in immunopathogenesis. Blood (2010) 116(4):515–21. 10.1182/blood-2010-01-266668 20339090

[22] Harris DP Haynes L Sayles PC Duso DK Eaton SM Lepak NM. Reciprocal regulation of polarized cytokine production by effector B and T cells. Nat Immunol (2000) 1(6):475–82. 10.1038/82717 11101868

[23] Luu VP Vazquez MI Zlotnik A. B cells participate in tolerance and autoimmunity through cytokine production. Autoimmunity (2014) 47(1):1–12. 10.3109/08916934.2013.856006 24245950

[24] Rinaldi A Chiaravalli AM Mian M Zucca E Tibiletti MG Capella C. Serotonin Receptor 3A Expression in Normal and Neoplastic B Cells. Pathobiology (2010) 77(3):129–35. 10.1159/000292646 20516728

[25] Akdis CA Simons FE. Histamine receptors are hot in immunopharmacology. Eur J Pharmacol (2006) 533(1-3):69–76. 10.1016/j.ejphar.2005.12.044 16448645

[26] D’Mello C Almishri W Liu H Swain MG. Interactions Between Platelets and Inflammatory Monocytes Affect Sickness Behavior in Mice With Liver Inflammation. Gastroenterology (2017) 153(5):1416–28.e2. 10.1053/j.gastro.2017.08.011 28802564

[27] Davis RP Almishri W Jenne CN Swain MG. The Antidepressant Mirtazapine Activates Hepatic Macrophages, Facilitating Pathogen Clearance While Limiting Tissue Damage in Mice. Front Immunol (2020) 11:578654(2839). 10.3389/fimmu.2020.578654 33250892 PMC7673391

[28] Haas KM Poe JC Steeber DA Tedder TF. B-1a and B-1b Cells Exhibit Distinct Developmental Requirements and Have Unique Functional Roles in Innate and Adaptive Immunity to S. pneumoniae. Immunity (2005) 23(1):7–18. 10.1016/j.immuni.2005.04.011 16039575

[29] Haas KM. B-1 lymphocytes in mice and nonhuman primates. Ann New York Acad Sci (2015) 1362(1):98–109. 10.1111/nyas.12760 25930711 PMC4627897

[30] Haas KM Poe JC Tedder TF. CD21/35 promotes protective immunity to Streptococcus pneumoniae through a complement-independent but CD19-dependent pathway that regulates PD-1 expression. J Immunol (2009) 183(6):3661–71. 10.4049/jimmunol.0901218 PMC3717971 19710450

[31] Li H Zhang G-X Chen Y Xu H Fitzgerald DC Zhao Z. CD11c+CD11b+ dendritic cells play an important role in intravenous tolerance and the suppression of experimental autoimmune encephalomyelitis. J Immunol (Baltimore Md 1950) (2008) 181(4):2483–93. 10.4049/jimmunol.181.4.2483 PMC2676731 18684939

[32] Hirota SA Ng J Lueng A Khajah M Parhar K Li Y. NLRP3 inflammasome plays a key role in the regulation of intestinal homeostasis. Inflammation Bowel Dis (2011) 17(6):1359–72. 10.1002/ibd.21478 PMC3026862 20872834

[33] Davis RP Surewaard BGJ Turk M Carestia A Lee W-Y Petri B. Optimization of In vivo Imaging Provides a First Look at Mouse Model of Non-Alcoholic Fatty Liver Disease (NAFLD) Using Intravital Microscopy. Front Immunol (2020) 10:2988. 10.3389/fimmu.2019.02988 31969883 PMC6960139

[34] Xu W Joo H Clayton S Dullaers M Herve M-C Blankenship D. Macrophages induce differentiation of plasma cells through CXCL10/IP-10. J Exp Med (2012) 209(10):1813–S2. 10.1084/jem.20112142 PMC3457728 22987802

[35] Dhirapong A Lleo A Yang G-X Tsuneyama K Dunn R Kehry M. B cell depletion therapy exacerbates murine primary biliary cirrhosis. Hepatology (2011) 53(2):527–35. 10.1002/hep.24044 PMC3058242 21274873

[36] Vigano M Mangia G Lampertico P. Management of patients with overt or resolved hepatitis B virus infection undergoing rituximab therapy. Expert Opin Biol Ther (2014) 14(7):1019–31. 10.1517/14712598.2014.912273 24909454

[37] Klinker MW Lundy SK. Multiple mechanisms of immune suppression by B lymphocytes. Mol Med (2012) 18(1):123–37. 10.2119/molmed.2011.00333 PMC3276396 22033729

[38] Shimomura Y Mizoguchi E Sugimoto K Kibe R Benno Y Mizoguchi A. Regulatory role of B-1 B cells in chronic colitis. Int Immunol (2008) 20(6):729–37. 10.1093/intimm/dxn031 18375938

[39] Yang YQ Yang W Yao Y Ma HD Wang YH Li L. Dysregulation of peritoneal cavity B1a cells and murine primary biliary cholangitis. Oncotarget (2016) 7(19):26992–7006. 10.18632/oncotarget.8853 PMC5053627 27105495

[40] Frolkis AD Vallerand IA Shaheen AA Lowerison MW Swain MG Barnabe C. Depression increases the risk of inflammatory bowel disease, which may be mitigated by the use of antidepressants in the treatment of depression. Gut (2019) 68(9):1606–12. 10.1136/gutjnl-2018-317182 30337374

[41] Stein JV Nombela-Arrieta C. Chemokine control of lymphocyte trafficking: a general overview. Immunology (2005) 116(1):1–12. 10.1111/j.1365-2567.2005.02183.x 16108812 PMC1802414

[42] Wang H Beaty N Chen S Qi CF Masiuk M Shin DM. The CXCR7 chemokine receptor promotes B-cell retention in the splenic marginal zone and serves as a sink for CXCL12. Blood (2012) 119(2):465–8. 10.1182/blood-2011-03-343608 PMC3257011 22110250

[43] Drennan MB Franki AS Dewint P Van Beneden K Seeuws S van de Pavert SA. Cutting edge: the chemokine receptor CXCR3 retains invariant NK T cells in the thymus. J Immunol (2009) 183(4):2213–6. 10.4049/jimmunol.0901213 19620294

[44] Ma Q Jones D Springer TA. The chemokine receptor CXCR4 is required for the retention of B lineage and granulocytic precursors within the bone marrow microenvironment. Immunity (1999) 10(4):463–71. 10.1016/s1074-7613(00)80046-1 10229189

[45] Lee W-Y Moriarty TJ Wong CHY Zhou H Strieter RM van Rooijen N. An intravascular immune response to Borrelia burgdorferi involves Kupffer cells and iNKT cells. Nat Immunol (2010) 11(4):295–302. 10.1038/ni.1855 20228796 PMC5114121

[46] Curbishley SM Eksteen B Gladue RP Lalor P Adams DH. CXCR 3 activation promotes lymphocyte transendothelial migration across human hepatic endothelium under fluid flow. Am J Pathol (2005) 167(3):887–99. 10.1016/S0002-9440(10)62060-3 PMC1698725 16127166

[47] Piali L Weber C LaRosa G Mackay CR Springer TA Clark-Lewis I. The chemokine receptor CXCR3 mediates rapid and shear-resistant adhesion-induction of effector T lymphocytes by the chemokines IP10 and Mig. Eur J Immunol (1998) 28(3):961–72. 10.1002/(sici)1521-4141(199803)28:03<961::Aid-immu961>3.0.Co;2-4 9541591

[48] Ruddell RG Mann DA Ramm GA. The function of serotonin within the liver. J Hepatol (2008) 48(4):666–75. 10.1016/j.jhep.2008.01.006 18280000

[49] Tilg H Kaser A Moschen AR. How to modulate inflammatory cytokines in liver diseases. Liver Int (2006) 26(9):1029–39. 10.1111/j.1478-3231.2006.01339.x 17032402

[50] Schmidt-Arras D Rose-John S. IL-6 pathway in the liver: From physiopathology to therapy. J Hepatol (2016) 64(6):1403–15. 10.1016/j.jhep.2016.02.004 26867490

[51] Mössner R Lesch KP. Role of serotonin in the immune system and in neuroimmune interactions. Brain Behav Immun (1998) 12(4):249–71. 10.1006/brbi.1998.0532 10080856

[52] Iken K Chheng S Fargin A Goulet AC Kouassi E. Serotonin upregulates mitogen-stimulated B lymphocyte proliferation through 5-HT1A receptors. Cell Immunol (1995) 163(1):1–9. 10.1006/cimm.1995.1092 7758118

[53] Anttila SA Leinonen EV. A review of the pharmacological and clinical profile of mirtazapine. CNS Drug Rev (2001) 7(3):249–64. 10.1111/j.1527-3458.2001.tb00198.x PMC6494141 11607047

[54] Wan M Ding L Wang D Han J Gao P. Serotonin: A Potent Immune Cell Modulator in Autoimmune Diseases. Front Immunol (2020) 11:186. 10.3389/fimmu.2020.00186 32117308 PMC7026253

[55] Choquet D Korn H. Dual effects of serotonin on a voltage-gated conductance in lymphocytes. Proc Natl Acad Sci USA (1988) 85(12):4557–61. 10.1073/pnas.85.12.4557 PMC280470 3260036

[56] Wąsik M Nazimek K Bryniarski K. Regulatory B cell phenotype and mechanism of action: the impact of stimulating conditions. Microbiol Immunol (2018) 62(8):485–96. 10.1111/1348-0421.12636 29998521

