# Supplementary material for: The Antidepressant Mirtazapine Rapidly Shifts Hepatic B Cell Populations and Functional Cytokine Signatures in the Mouse
Source: Front Immunol. 2021 Mar 25;12:622537. doi: 10.3389/fimmu.2021.622537 (PMC8027111; doi:10.3389/fimmu.2021.622537)
Supplement: Supplementary file 1 [file DataSheet_1.pdf]

Supplementary Material

1 Supplementary Data to:

The antidepressant mirtazapine induces a rapid shift in hepatic B cell populations and associated functional cytokine signatures in the mouse

Wagdi Almishri, Rachelle P Davis, Abdel-Aziz Shaheen, Mohammed O. Altonsy, Craig N Jenne, Mark G Swain

Table of Contents

Table of Contents ………………………….……………………….……1

Supplemental Figuer1 ……………………………………...……………… 2

Supplemental Figuer2 ……………………………………………………... 3

Supplemental Figuer3 ……………………………………………………... 4

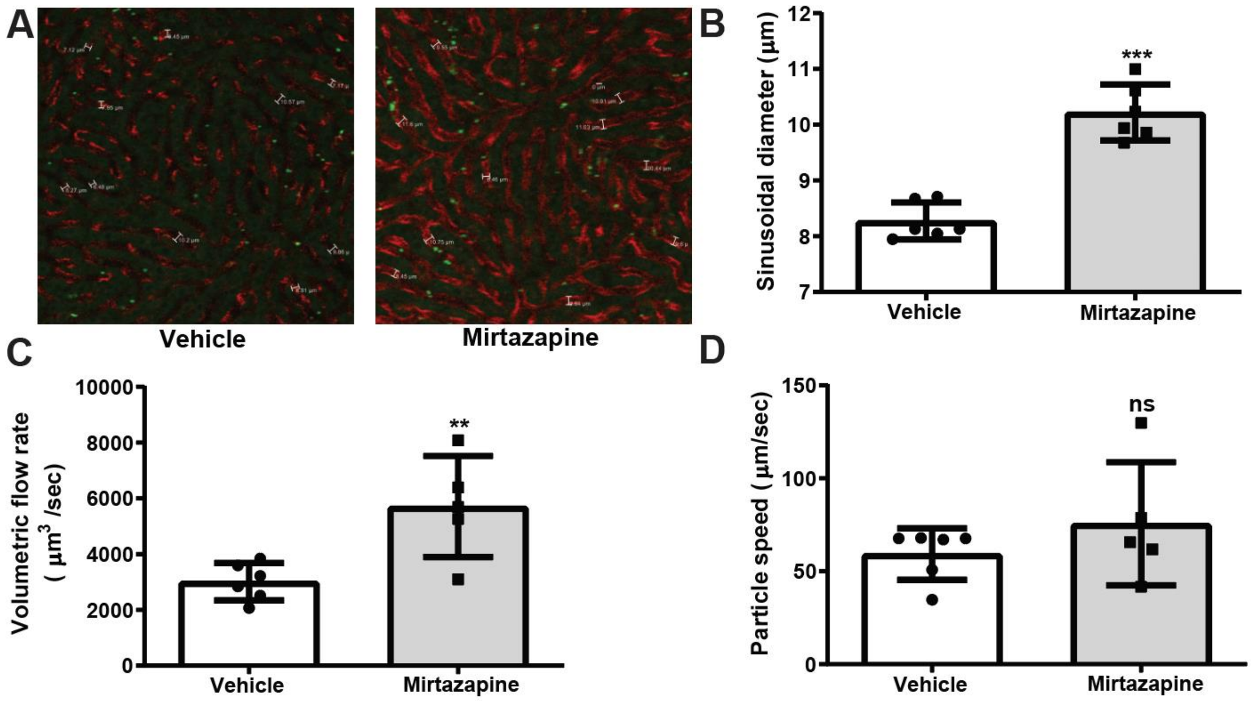

Supplementary Figure 1. Mirtazapine-induced alterations in the hepatic microcirculation.

Mice were injected with mirtazapine or vehicle and changes in sinusoidal diameter and the volumetric flow rate in liver sinusoids measured 3 hrs later. (A) Representative IVM images showing liver sinusoids and diameter measurements in vehicle and mirtazapine treated mice. Vessels were perfused with contrast agent (rhodamine dextran) to enhance visualization. (B) Quantification of IVM images showing increased sinusoidal diameter, and (C) flow rate, in mice treated with mirtazapine compared to vehicle treated controls. Particle speed (D) was not statistically different (n = 5 mice per group). Data are shown as mean +/- SEM, Student's t test (**$p \leq 0.0001$, ***$p \leq 0.008$, n = 5 and 6 mice/group).

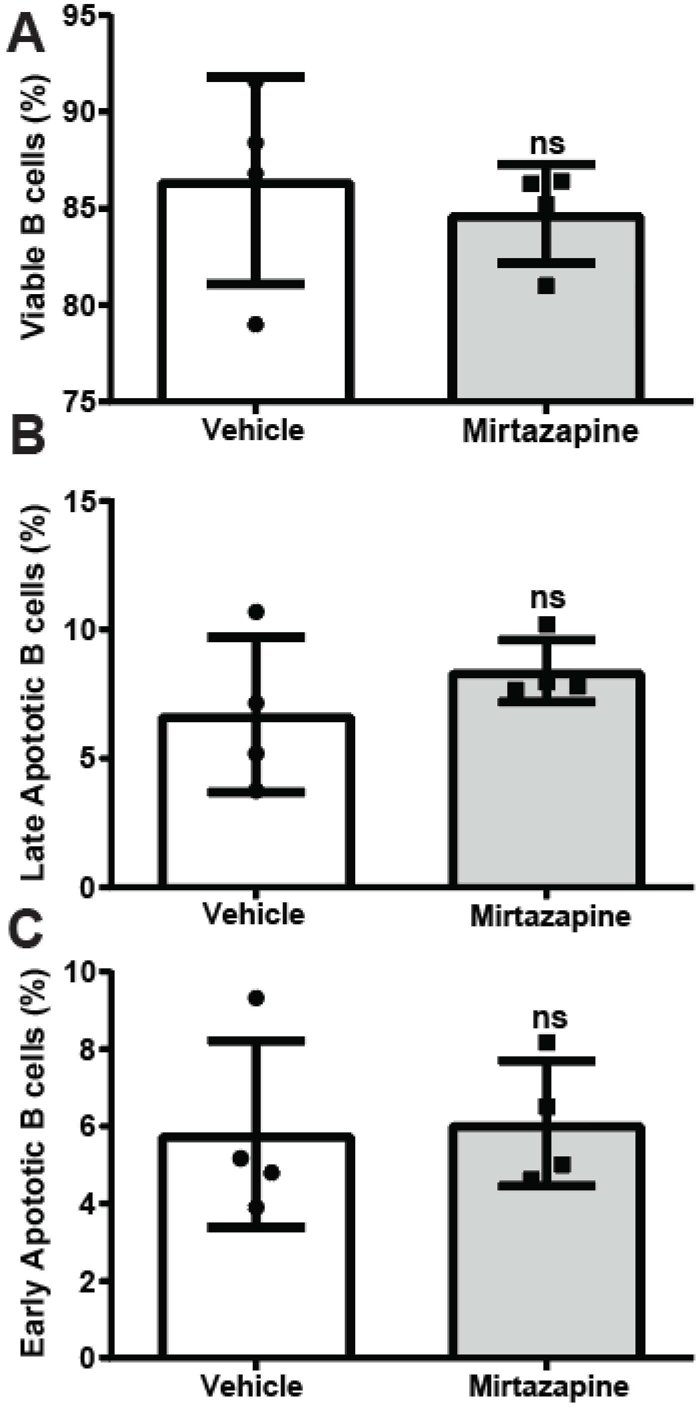

Supplementary Figure 2. Mirtazapine has no direct apoptotic or cytotoxic effect on B cells.

To evaluate whether mirtazapine has any apoptotic or cytotoxic effect on B cells, whole blood was treated with mirtazapine (10 µM) or vehicle for 4 h. The cell viability and cell death of B cells was evaluated by flow cytometry. Mirtazapine has no direct toxicity on B cells at 10 µM as cell viability (A) and markers of apoptosis (B and C) remained unchanged after incubating with mirtazapine (n = 4 for per group).

.

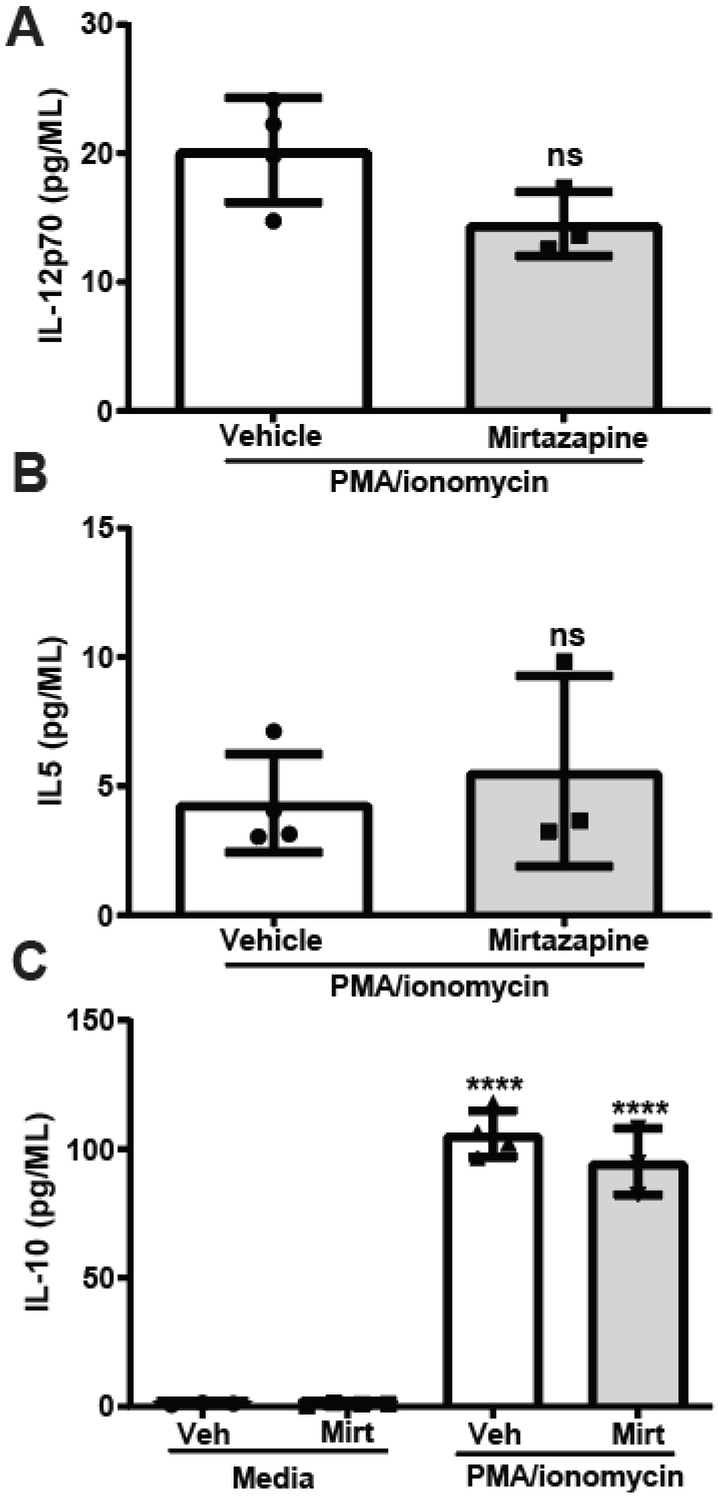

Supplementary Figure 3. In vivo treatment with mirtazapine did not later hepatic B cells capacity to produce IL12 p70, IL5, and IL10.

Hepatic CD19$^+$ cells (isolated from vehicle or mirtazapine-treated mice) were stimulated with PMA/ionomycin and the release of the cytokines IL12 p70, IL-5, and IL-10 were measured in cell culture supernatants. Mirtazapine did not alter hepatic B cell stimulated production of the cytokines IL12p70 (A), IL-5 (B), and IL-10 (C) (n = 3 and 4 for per group). IL-12p70 and IL-5 cytokines were below detection levels in media-treated control wells.
